# THORACOLOMBAR BURST FRACTURES: SHORT FIXATION, WITHOUT ARTHRODESIS AND WITHOUT REMOVAL OF THE IMPLANT

**DOI:** 10.1590/1413-785220233101e253655

**Published:** 2023-04-17

**Authors:** Carlos Humberto Targa Moreira, Walter Krause, Robert Meves

**Affiliations:** 1 Universidade da Santa Casa de São Paulo, Department of Orthopedics and Traumatology, São Paulo, SP, Brazil.; 2 Instituto de Ortopedia e Traumatologia, Dourados, MS, Brazil.; 3 Universidade São Judas Tadeu, Department of Physical Education, Laboratory of Morphoquantitative and Immunohistochemistry Studies, São Paulo, SP, Brazil.

**Keywords:** Accidents, Thoracic Vertebrae, Lumbar Vertebrae, Arthrodesis, Acidentes, Vértebras Torácicas, Vértebras Lombares, Artrodese

## Abstract

**Objectives:**

To present the functional outcomes, through the first case series in our country, of patients with thoracolumbar burst fractures (A3,A4), submitted to short posterior fixation, without arthrodesis and without removal of the implants, until the end of the minimum follow-up of one year.

**Methods:**

Fifty five patients consecutively treated between January/2010 and January/2019 were evaluated through medical records and imaging exams. Radiographic analysis was performed by mea suring local and segmental kyphosis using the Cobb method. Functional assessment was analyzed using the non-specific SF-36 questionnaire and the 1983 Denis pain and work-specific questionnaire, applied after 12 months of follow-up.

**Results:**

With a loss of five patients (9%), 22 (44%) patients reported having minimal and occasional pain and 8 (16%) patients reported having no pain. Three (6%) patients responded that they were completely incapacitated. Patients had a mean score of 73.16 points in the SF-36 domains. There was a significant reduction in kyphosis in 12 months (9.1±5.2 [min-max 0-22]) compared to the preoperative period (14.9±7.8 [min-max 0-32]) ( p≤0.01). One patient required implant removal due to the symptomatic prominence of the implant.

**Conclusion:**

This case series suggests that the technique leads to satisfactory functional results, without implant failure or significant kyphosis after a minimum follow-up of 12 months of treatment. ***Evidence Level IV; Case series.***

## RESUMO

**Objetivo:**

Apresentar os desfechos funcionais, mediante primeira série de casos no nosso meio, de pacientes com fratura toracolombar do tipo explosão (A3, A4), submetidos a fixação posterior curta, sem artrodese e sem retirada dos implantes, até o final do acompanhamento mínimo de um ano.

**Métodos:**

Foram avaliados, por meio de prontuários e exames de imagem, 55 pacientes consecutivamente tratados entre Janeiro/2010 e Janeiro/2019. A análise radiográfica foi realizada medindo a cifose local e segmentar, pelo método de Cobb. A avaliação funcional analisada por meio do questionário inespecífico SF-36 e questionário específico de dor e trabalho de Denis de 1983, aplicados após os 12 meses de seguimento.

**Resultados:**

Com perda de cinco pacientes (9%), 22 (44%) pacientes relataram ter dor mínima e ocasional e 8 (16%) pacientes responderam não ter dor. Três (6%) pacientes responderam que estavam completamente incapacitados. Os pacientes tiveram uma pontuação média de 73,16 pontos nos domínios do SF-36. Houve redução significativa da cifose em 12 meses (9,1±5,2 [min-máx 0-22]) na comparação com o pré-operatório (14,9±7,8 [min-máx 0-32]) (p≤0,01). Um paciente necessitou de retirada do implante em razão da proeminência sintomática do implante.

**Conclusão:**

Esta série de casos sugere que a técnica leva a resultados funcionais satisfatórios, sem falha do implante ou cifose pós-traumática após acompanhamento mínimo de 12 meses de tratamento. ***Nível de Evidência IV; Série de casos.***

## INTRODUCTION

The thoracolumbar transition is most affected by fractures of the spine as it is a region located between the less flexible thoracic spine and the more flexible lumbar spine.^1^

Burst fractures are one of the most common types of fractures of the thoracolumbar spine, mainly caused by trauma mechanisms in the axial direction, ranging from 21 to 64% of all thoracolumbar fractures.^2^ Studies show that the short posterior fixation with screws in the fracture is enough to obtain stability, avoiding the need for instrumented fusion of the long segment.^3,4^ However, there still seems to be no consensus regarding the long-term results.^5^

Despite the development of biomechanical studies and new instrumentation systems, clinical studies still discuss the most appropriate surgical procedure to treat burst fractures since ventral, dorsal and combined instrumentation are available,^5^ long or short fixation, with or without intermediate screw.

Furthermore, the fusion or arthrodesis of the fixed segment may or may not be performed, even if there are studies that demonstrate that there is no clinical and radiographic difference in the results.^2^ In view of the lack of consensus and the scarcity of studies on fixation without arthrodesis and without removing implants in our country, we proposed a series of cases with short fixation without arthrodesis to analyze the clinical and radiographic outcomes, with a minimum follow-up of one year.

Therefore, the aim of this study was to analyze the functional and radiographic outcomes of patients with thoracolumbar burst fractures according to AO (A3/A4), treated with short fixation via the posterior approach, without arthrodesis and without removal of the implants until the end of the minimum follow-up of 12 months.

## MATERIALS AND METHODS

This study was approved by the Research Ethics Committee of Santa Casa de São Paulo, under protocol number 2.573.255, and by the Management Committee for Internships, Projects, Research, Extensions and Work–CGEPET, from the Dourados Health Services Foundation – FUNSAUD, by letter of authorization. All patients included in this study read and signed an informed consent form. Of a total of 55 patients, five lost follow-up during follow-up. Therefore, 50 patients completed the study (38 males and 12 females). The mean follow-up period for patients was 14.5±12.4 months (ranging from 12 to 60 months).

This study is a retrospective analysis of consecutive case series, using medical records and imaging exams (x-ray [XR] and computed tomography [CT]), with data from pre- and postoperative assessments of patients with thoracolumbar burst fractures type A3 and A4, according to the AOSpine classification.^6,7^ Patients without neurological deficit underwent short posterior fixation, without arthrodesis, with a screw in the fractured vertebra, according to the technique described by Kanna et al.^8^

Inclusion criteria were: thoracolumbar burst fractures classified as A3 and A4 by AOSpine, without neurological deficit, acute, which underwent surgical treatment with short fixation, including the fractured vertebra, without arthrodesis.

Exclusion criteria were: multiple-level fractures, fractures caused by other diseases, fractures not classified as A3 or A4, cases with arthrodesis or with a larger fixed segment, patients with neurological deficit, patients with less than one year of follow-up, incomplete medical record, lack of radiographs at the beginning and end of follow-up, loss of follow-up or non-acceptance to participate in the study.

The variables studied were age, gender, level affected, pre- and postoperative Cobb angles (1948) and neurological status according to the Scale by Frankel et al (1969).^9,10^

The primary functional outcomes evaluated in this study were pain and postoperative functional capacity, according to the criteria of Denis (1983). As a secondary outcome, quality of life was assessed according to the SF-36 validated for the Portuguese language.^11,12^ The patients underwent functional analysis based on the Denis Pain and Work Scale^13^ and the Short–Form 36 (SF-36) quality of life questionnaire applied at the last follow-up.

**Figure 1:**
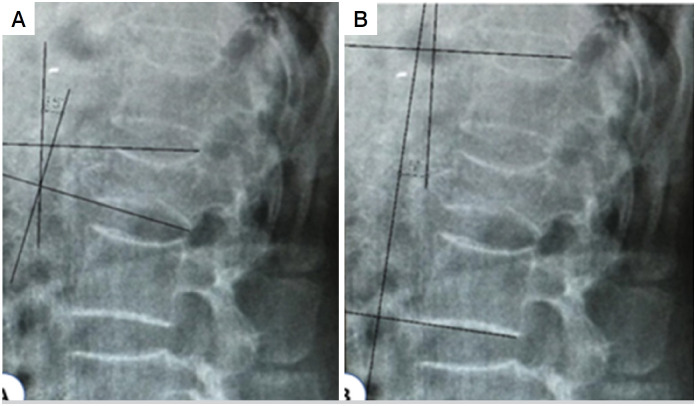
Illustration of the Cobb angle (A - local kyphosis 15 degrees and B- segmental kyphosis 5 degrees).

Radiographic analysis was performed by measuring local and segmental kyphosis using the Cobb method.^9,14^ To measure the segmental kyphosis (fixed segment), immediately preserved endplates, cranial and caudal at the affected level were used as reference. For local kyphosis (fractured vertebra), the end plates of the upper and lower fractured vertebrae were used for the measurement (Figure 1), according to the methodology applied by Siebenga et al.^15^ The measurement was performed on lateral view radiographs performed on three occasions: at the initial diagnosis (baseline), in the immediate postoperative period (immediate) and at the end of the 12-month follow-up.

### Surgical technique

With the patients under general anesthesia and positioned in the prone position, a standard posterior midline incision was made. Using the technique of Kanna et al,^8^ we started with the classic posterior exposure of the spine through the midline. The posterior ligament complex and the facet joint were preserved during the exposure.

**Figure 2:**
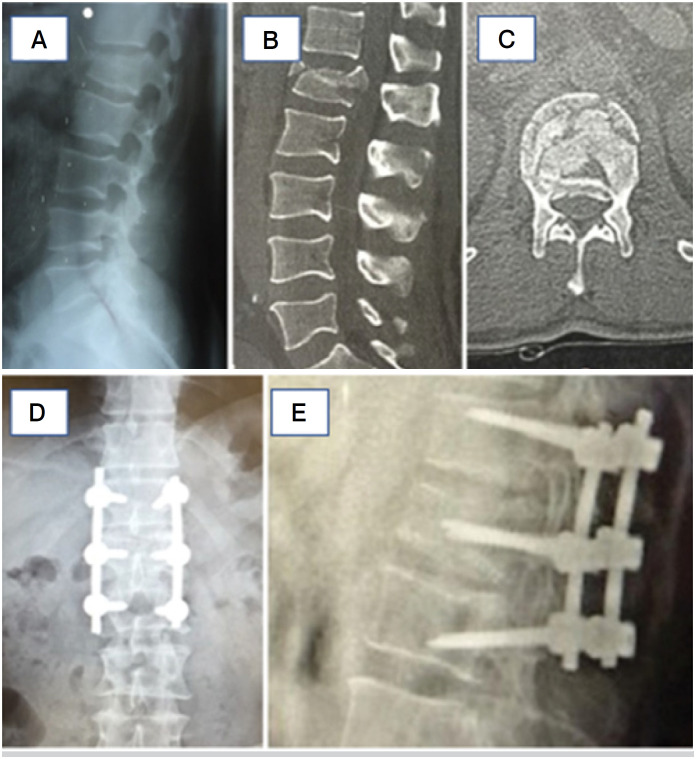
Illustrative images of the pre (A, B, and C) and postoperative (D and E) periods of 12 months. A- Preoperative x-ray; B- Sagittal plane computed tomography; C- Axial plane computed tomography; D- Post-op- erative anteroposterior x-ray 12 months; E- Posterior post-operative X-ray 12 months.

The screw entry point in the pedicle was located, using the technique of Roy-Camille et al (1986), at the junction point of the transverse process and upper facet with a pointed perforator.^16^ We used intraoperative radioscopy and polyaxial screws in all procedures. The placement of screws in the vertebrae above and below the fracture was started, with divergent inclination (Figure 2), so that when placing the longitudinal bars lightly molded in lordosis, bilateral distraction of the fracture was made, reducing the wedging of the fractured vertebra and the local and segmental kyphosis. The fractured vertebra was also screwed bilaterally.

Through the ligamentotaxis mechanism, the reduction and decompression of the vertebral canal was carried out indirectly.^1^ Thus, the reduction procedure included the following steps: improvement of the local and segmental kyphosis, lordotic distraction to reduce the vertebral height and the intra-canal fragment, and rigid block with the placement of counter screws.^17^ The implants, in theory, should be removed after radiographic consolidation, which occurs on average six months after the fracture.

### Statistical analysis

For the final analysis of the data, descriptive statistics (mean, standard deviation, median and minimum-maximum values) were used.

To determine the normality of the sample, we used the Shapiro-Wilk test. As most of the variables did not have a normal distribution, non-parametric tests were chosen.

To compare the segmental and local kyphosis angles, the Friedman's non-parametric test was used. For comparison between the evaluated moments, the Wilcoxon test was applied. For the correlation between the Denis Pain and Work Scale scores and the SF-36 quality of life questionnaire, Spearman's correlation test was used.

The SPSS version 13.0 program was used to perform the statistical analysis of this work. The significance value was set at p≤0.05.

## RESULTS

Following the criteria for inclusion of fractures, 39 cases were classified as type A3 (78%) and 11 cases were classified as type A4 (22%).

### Pain and Work Scale

On the Denis Pain Scale, 22 patients (44%) reported minimal and occasional pain. Moderate pain was reported by 20 (40%) patients, but without interruption of daily activities or work. Other patients (16%) responded that they were without pain after one year of follow-up. (Table 1)

**Table 1:** Description of the frequency of responses regarding the Pain and Work scale of Denis (1983).

Criteria	Frequency (nº)	Percentage (%)
**Pain Scale**		
D1	8	16
D2	22	44
D3	20	40
D4	0	0
D5	0	0
Total	50	100
**Work Scale**		
T1	12	24
T2	24	48
T3	11	22
T4	0	0
T5	3	6
Total	50	100

On the Denis Work Scale, 24 patients (48%) responded that they would be able to return to work (sedentary) or heavy work with restrictions. In addition to these, 12 (24%) patients responded that they felt able to return to previous work (heavy) or heavy physical activities. However, 11 (22%) patients reported that they felt unable to return to their previous job but were working full time in a new job, and only three (6%) patients responded that they were completely disabled. (Table 1)

### Evaluation of Segmental and Local Kyphosis

**Figure 3:**
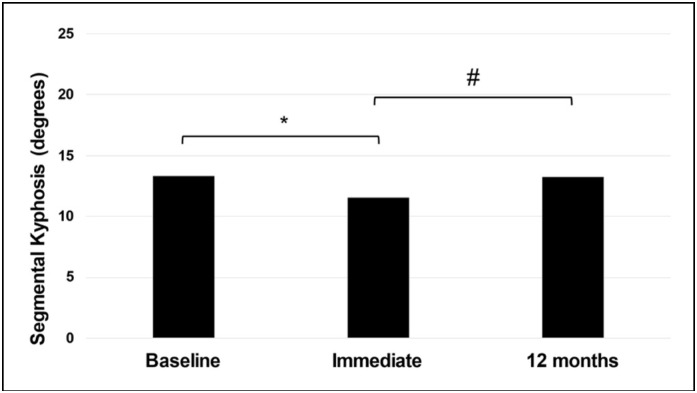
Comparison of the degrees of segmental kyphosis between the preoperative (Baseline), immediate postoperative (immediate), and 12 months postoperative (12 months) moments. Legend:* statistically different from baseline; #statistically different than 12 months.

The analysis of segmental kyphosis showed a statistical difference between the evaluated moments (p≤0.01). There was a smaller angle of segmental kyphosis in the immediate post-surgical moment (11.5±8.7 [min-max 0-31]) compared to the baseline moment (13.3±8.5 [min-max 0] -40]) and 12 months (13.2±7.8 [min-max 2-36]) (p≤0.01, Figure 3).

**Figure 4:**
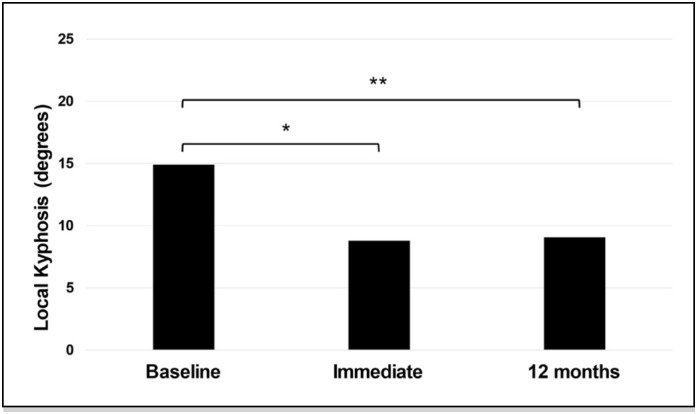
Comparison of degrees of local kyphosis between preoperative (Baseline), immediate postoperative (immediate), and 12 months post-operatively (12 months). Legend:* statistically different from immediate; **statistically different from 12 months.

The analysis of local kyphosis showed a statistical difference between the moments (p≤0.01), with a significant reduction in the angulation in the immediate postoperative period (8.8±5.4 [minmax 1-22]) and 12 months ( 9.1±5.2 [min-max 0-22]) compared to baseline (14.9±7.8 [min-max 0-32]) (p≤0.01, Figure 4).

One patient underwent removal of the synthesis due to pain and local discomfort in the prominence of the implant and reported, after three months of removal of the synthesis, an improvement in local pain and movement.

There were no cases of infection, implant failure, neurological deficit, or significant loss of correction until the end of follow-up.

### Quality of Life Assessment

At the end of 12-month follow-up, patients scored above 70 in all SF-36 domains, with the exception of physical limitation (48.5±38.3) and pain (69.8±14). (Table 2)

**Table 2:** Description of the mean, median, standard deviation, and minimum-maximum values of the domains analyzed by the SF-36 questionnaire.

Domains	Mean	Median	Minimum	Maximum
Social Aspects	89.4	100.0	25.0	100.0
Mental Health	80.7	80.0	28.0	100.0
Emotional Limitations	75.9	100.0	0.0	100.0
Vitality	74.9	75.0	30.0	100.0
Functional capacity	74.1	75.0	15.0	100.0
General Health Status	72.0	72.0	52.0	100.0
Pain	69.8	74.0	30.0	100.0
Physical Limitation	48.5	50.0	0.0	100.0

**Figure 5:**
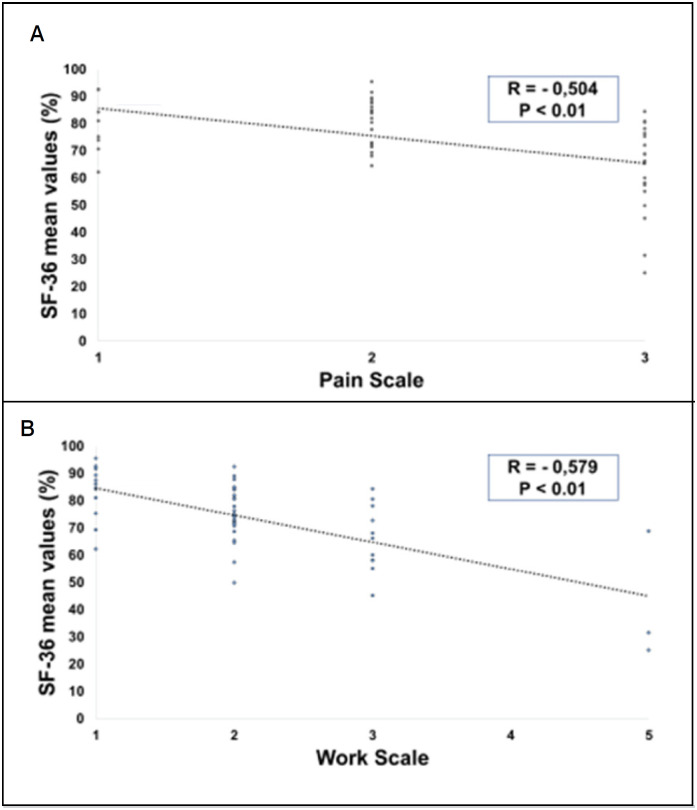
Spearman correlation coefficient tests between Denis pain (A) and work (B) scale scores and general SF-36 questionnaire domains.

Correlation tests demonstrated an inverse relationship between the Denis Pain and Work Scale scores and many SF-36 domains. (Figure 5)

Regarding the eight domains evaluated in the SF-36 (Table 3), significant correlations were found between DD and CF, LF, DOR, EGS, AS and LE. Regarding TD, significant correlations were found with all SF-36 domains.

**Table 3:** Spearman correlation coefficient tests between Denis pain [DP] and work [DW] scale scores and the SF-36 questionnaire domains (functional capacity [FC], physical limitation [PL], pain, general health status [GHH], vitality [VITA], social aspects [SA], emotional limitations [EL] and mental health [MH]).

		FC	PL	PAIN	GHH	VITA	SA	EL	MH
DP	Correlation	-0.634	-0.318	-0.632	-0.373	-0.259	-0.429	-0.292	-0.169
	P-value	0.000	0.025	0.000	0.008	0.069	0.002	0.040	0.241
DW	Correlation	-0.552	-0.319	-0.490	-0.460	-0.436	-0.417	-0.376	-0.465
	P-value	0.000	0.024	0.000	0.001	0.002	0.003	0.007	0.001

## DISCUSSION

Few studies have compared different treatments in a target population with neurologically intact A3 and A4 fractures.^18^ According to Rometsch et al.,^18^ although previous studies have shown a possible benefit of surgical treatment for fractures of types A3 and A4, there is no consensus. However, it is still difficult to discuss parts of these outcomes due to the great variability of measurement techniques. Furthermore, most studies do not differentiate fractures into incomplete (A3) and complete (A4).^18^

In this study, it was shown that after one year of follow-up, 84% of patients reported mild and moderate pain, and 72% said they would return to work despite the small loss of the final segmental kyphosis. Recently, Özbek et al.^19^ reported that short fixation had better clinical results and faster fusion despite the loss of the final postoperative kyphosis. The studies corroborate our findings, whose segmental kyphosis decreased soon after surgery, despite the return to baseline values after 12 months. ^19-21^ In the study by Chou et al.,^19^ the loss of segmental kyphosis was attributed to the loss of height of the injured intervertebral disc and not the progression of kyphosis in the fracture. These findings corroborate the results of this study, as local kyphosis remained practically stable for up to 12 months. Furthermore, despite the small loss in the final segmental kyphosis, there was no compromise in the clinical and functional results.

By analyzing the literature, studies have shown that there is no significant difference between treatments, with and without arthrodesis, after fixation of burst fractures with pedicle screws.^22,21,02^ However, few studies have described the surgical treatment of thoracolumbar burst fractures using the posterior short fixation technique, including the fractured vertebra and without arthrodesis.^22,08,17,23^ Our study adds information on outcomes without removing the synthesis material, which is routinely recommended for this technique after fracture healing. In our environment, it is argued that the vast majority of spinal surgery services do not follow the recommendation for removal of implants. The main reasons are the logistical difficulty for an elective vacancy and the non-adherence of the indication for removal of implants in asymptomatic patients. The observation of our patients showed that even without the removal of the implant in 49 patients, there was no case of infection, implant failure, neurological deficit or loss of the final significant correction until the end of follow-up.

Wang et al. reported satisfactory results without fusion. However, the authors did not classify fractures as A3 and A4.^22^ Furthermore, Kanna et al., in a retrospective analysis, were also not limited to fractures in A3 and A4, including patients with neurological deficit.^8^

Reinforcing our results, Zhao et al. reported that the technique with intermediate screws can prevent the loss of postoperative kyphosis in patients without neurological deficit, as demonstrated here.^17^ Finally, Liao and Fan described a group of patients with type A3 fractures, operated with short fixation, including the fractured vertebra and without arthrodesis, as in our study, but they did not include type A4 fractures, as we did.^23^ As in this study, the final clinical results were evaluated by the Denis Pain and Work Scales,^13^ in addition to the kyphotic angle by the Cobb method. The mean pain and work score was 1.5 ± 0.8 and 1 .7 ± 0.9, respectively. Segmental kyphosis significantly reduced after surgery, with little loss at the end of follow-up. Regarding the results of the Denis pain questionnaire,^13^ it was found that patients answered D1 (no pain), D2 (occasional) and D3 (moderate) scores, characterizing lesser complaints. The results are supported by the studies by Zhao et al. and Liao and Fan.^17,23^ When making the same comparison with Denis’ work questionnaire scores, a pattern similar to those cited by Liao and Fan was observed, characterizing better ability to return to previous work.^23^ When comparing the quality of life results of our operated patients, after 12 months of follow-up, with the results of Laguardia et al., from the normal population, better results were observed after surgery, suggesting a significant improvement in pain and mobility.^24^ This demonstrates the assumption that the surgical procedure described was little disabling from a physical point of view.

Finally, this study has some methodological limitations. First, lack of a control group. Second, a greater number of assessments at baseline and postoperatively would allow for a more accurate comparison of patients’ functional status and quality of life. Third, the follow-up period of just 12 months. And, finally, the fact that the evaluations were not applied blindly by the evaluator.

## CONCLUSION

Short fixation without arthrodesis and without removal of the implant via the posterior route of A3 and A4 burst-type thoracolumbar spine fractures seems to be an efficient method for spinal stabilization, enabling satisfactory clinical and radiographic results after a minimum follow-up of 12 months.
